# Longitudinal change in physical functioning and dropout due to death among the oldest old: a comparison of three methods of analysis

**DOI:** 10.1007/s10433-019-00533-x

**Published:** 2019-09-18

**Authors:** Jani Raitanen, Sari Stenholm, Kristina Tiainen, Marja Jylhä, Jaakko Nevalainen

**Affiliations:** 1 Faculty of Social Sciences (Health Sciences), Tampere University, PO Box 100, 33014 Tampere, Finland; 2 UKK Institute for Health Promotion Research, PO Box 30, 33501 Tampere, Finland; 3 Department of Public Health, University of Turku and Turku University Hospital, PO Box 52, 20521 Turku, Finland; 4 Centre for Population Health Research, University of Turku and Turku University Hospital, Turku, Finland; 5 Gerontology Research Center, Tampere University, PO Box 100, 33014 Tampere, Finland; 6 Science Center of Tampere University Hospital, PO Box 2000, 33521 Tampere, Finland

**Keywords:** Longitudinal study, Functioning, Attrition due to death, Model comparison, Joint model

## Abstract

Longitudinal studies examining changes in physical functioning with advancing age among very old people are plagued by high death rates, which can lead to biased estimates. This study was conducted to analyse changes in physical functioning among the oldest old with three distinct methods which differ in how they handle dropout due to death. The sample consisted of 3992 persons aged 90 or over in the Vitality 90+ Study who were followed up on average for 2.5 years (range 0–13 years). A generalized estimating equation (GEE) with independent ‘working’ correlation, a linear mixed-effects (LME) model and a joint model consisting of longitudinal and survival submodels were used to estimate the effect of age on physical functioning over 13 years of follow-up. We observed significant age-related decline in physical functioning, which furthermore accelerated significantly with age. The average rate of decline differed markedly between the models: the GEE-based estimate for linear decline among survivors was about one-third of the average individual decline in the joint model and half the decline indicated by the LME model. In conclusion, the three methods yield substantially different views on decline in physical functioning: the GEE model may be useful for considering the effect of intervention measures on the outcome among living people, whereas the LME model is biased regarding studying outcomes associated with death. The joint model may be valuable for predicting the future characteristics of the oldest old and planning elderly care as life expectancy continues gradually to rise.

## Introduction

Loss of physical functioning is a marker of declining health, and with time it seriously threatens older people’s independence and quality of life (Guralnik et al. 2000). Those with greater physical disability need more social and health care services and are less likely to remain active in the community and exhibit higher mortality rates (Guralnik et al. 1994; Hirvensalo et al. 2000).

Reliable information on changes in physical functioning with advancing age is needed for several purposes. For social policy and care planning, it is essential to have knowledge about the functioning of specific age cohorts at different ages, and researchers who want to understand the process of ageing will need to know how functioning changes with advancing age. Longitudinal studies with repeated measurements over time are necessary in order to produce accurate estimates of changes in functioning.

However, many longitudinal studies involving older adults face a critical limitation: some participants fail to provide data in at least some waves of the data collection (Jones et al. 2015). The most frequent reason for this is that participants have died or are too ill or weak to continue to take part. In studies concerned with functioning, these two reasons are interconnected: it is known that closeness of death is associated with poor health and declining functional status (Guralnik et al. 1991; Lunney et al. 2003). Therefore, in these cases it is likely that subjects who drop out are systematically different from those remaining in the study with respect to the outcomes of interest; they may have greater difficulties in functioning, for instance (Jones et al. 2015). Without the application of appropriate statistical methods, findings drawn from longitudinal studies may underestimate the decline in functioning, especially in the later stages of a study where ill health and death leave gaps in the data.

In statistical analysis, cases of missing data are commonly characterised as ‘missing completely at random’ (MCAR), ‘missing at random’ (MAR) or ‘missing not at random’ (MNAR). When data are MCAR, we assume that there is no relationship between the likelihood of a data point being missing and either unobserved or observed values. In MCAR conditions, the absence of data follows no particular pattern. In the case of MAR, we operate under the assumption that absence of data may exhibit a systematic relationship with the values for some observed variables but not with the missing values themselves. For example, respondents with lower education could be less likely to respond to a question on smoking. Since smoking tends to be more common in lower socio-economic classes, for instance, a simple overall estimate of smoking prevalence can yield a significant underestimate. Here, gaps can be addressed by using appropriate models or multiple imputation procedures if other characteristics of socio-economic class, such as income, are known (Rubin 1987; Little and Rubin 2002).

The most problematic form of missing data is MNAR. In these conditions, the probability of missing data depends on the unobserved values themselves; the missing values therefore differ systematically from the observed ones. For example, whether or not respondents supply data on their alcohol consumption may depend on whether their levels of consumption are excessive.

Death is a special kind of missing mechanism. There are fewer observations for those who have died than for survivors, but strictly speaking observations after death cannot be consider ‘missing’—they do not exist. However, in the absence of a better mechanism, MNAR would be the most appropriate way to handle dropout due to death.

Accounting for data points missing due to death is particularly important in studies among the oldest old, who exhibit high mortality rates. In their analysis of decline in functioning in Danish cohorts aged 92–100 years, Christensen et al. (2008) considered missing data due to non-participation by using an inverse probability weighting procedure. They rightly pointed out that correcting for non-response among survivors but not for that due to death is sufficient when the aim is to base the inference on the population still alive at each measurement point. They observed that the population-level decline in functioning across the measurements among surviving participants was negligible (compared with the steep decline at individual level) even when missing data correction was applied. The authors concluded that even if terminal illness was one of the main reasons for non-participation, the discrepancy between the individual and population trajectories was due primarily to deaths among the most disabled individuals and was not caused by non-participation.

In our study, we examined changes in physical functioning in a population aged 90 and over in a unique 13-year follow-up project called the Vitality 90+ Study. We applied three distinct methods: (i) generalized estimating equations (GEE) approach, which was used to estimate the longitudinal trend among survivors in a similar way as Christensen et al. (2008); (ii) a linear mixed-effects (LME) model, used as a reference method based on its extensive use in longitudinal data analysis; and (iii) a joint model which properly accounts for data points that are missing due to death. The first two methods were selected for their frequent use in longitudinal analyses of ageing, and the joint model because it can be used to address dropout due to death. Other methods not discussed here may be employed in place of the joint model, including selection models (e.g. Diggle and Kenward 1994) and pattern mixture models (e.g. Little 1993). Our aim was to compare the characteristics of the three methods when applied to data with high dropout rates due to death, and to see how quantitative estimates and their interpretations differ.

### The three ways of handling missing data in longitudinal studies

Several methods have been proposed to address the issue of missing data caused by death or other participant dropout in longitudinal studies (Dufouil et al. 2004; Biering et al. 2015; Moore et al. 2015). The development of physical functioning in the original study sample can differ considerably from that among members of the study sample who remain alive for each survey round. Levels of physical functioning tend to remain higher in the subgroup of survivors than among those who die between the measurement points, and this difference is only emphasised when mortality rates are high—say in research among very old people.

The idea behind the GEE approach, a common method of analysing longitudinal data (Liang and Zeger 1986), is simply to model the group-level (i.e. marginal) mean of the outcome—for example, physical functioning in a group of women or men. When measurements of physical functioning are conducted repeatedly over time, the data can also be viewed as a series of measurements made at fixed times. In GEE, the population-mean outcome is modelled at group level across these multiple cross sections, with the correlation between repeated observations being accounted for separately, to ensure valid inferences. The relationship between the outcome and covariates will thus be based only on survivors at each survey round. Thus, the GEE model treats the longitudinal outcomes as though they were cross sectional: depicting the average outcome among survivors on a given survey round rather than individuals’ trajectories (Kurland et al. 2009). Such a marginal model is useful when the investigator’s interest lies in examining sample-averaged effects, since the means being modelled are the averages for participants with the same covariate values.

The GEE approach is a valid method for complete data or when data are MCAR, but it may yield biased estimates in a MAR setting. When data are MAR, one may use reweighting methods (Robins et al. 1995), such as inverse probability weighted generalized estimating equations (IPW-GEE), which account for dropout by applying weights to each subject at each survey round and hence produce a valid inference. These weights can be derived for subjects still alive in each survey round by taking the inverse of the fitted probabilities of being alive in that round from a logistic regression model, which includes factors associated with death as explanatory variables. When death leads to missing data, direct estimation of how functioning develops among survivors can be easily obtained via GEE using an independent ‘working’ correlation structure. In the case of missing data due to death, non-independent structures will usually not yield valid inference. (Kurland and Heagerty 2005) With all data up to death included in the analysis, the GEE model estimates the longitudinal trend for a dynamic (i.e. changing) survivor cohort, not individuals (Kurland et al. 2009). Initially, the survivor cohort consists of every participant. However, after 1 year of follow-up, for example, it will comprise only those who survived the whole first year. As the survivor cohort gradually decreases in size, the GEE model can no longer describe the change in functioning for the whole initial cohort. Any other types of missing data (e.g. opting not to participate in one or more survey rounds) must be missing completely at random if the analysis is to remain valid (Dufouil et al. 2004).

Another commonly used means of analysing longitudinal data is an LME model, which includes both fixed and random effects. The mean outcome is determined on the basis of fixed effects, while between-subject heterogeneity and, thereby, the variance and within-subject correlation are modelled in terms of random effects (West et al. 2014). In an LME model, the longitudinal trajectory is modelled for the mean and for each subject. Such likelihood-based models can reliably estimate trajectories without access to complete data (Laird 1988). These models, however, have restricted applicability in that they can only deal with MAR-type incomplete datasets (Sterne et al. 2009; Neuhaus and McCulloch 2014). Models of this type are useful for modelling the longitudinal outcome if deaths either do not occur or are (conditionally) independent of the outcome process (Kurland et al. 2009). As closeness to death is likely to be associated with declining functioning (Guralnik et al. 1991; Lunney et al. 2003), LME models could provide biased assessments of developments related to functioning.

The third alternative for modelling longitudinal data is to use a joint modelling approach. This is based on simultaneous modelling of longitudinal and survival data. If the longitudinal outcome has a strong association with survival, fitting a joint model (also called a shared-parameter model) can reduce the bias that may result from non-random dropout due to death (Rizopoulos 2012). Joint estimation of a longitudinal and a survival model is achieved by assuming that they are correlated via subject-specific random effects (Rizopoulos 2010). The shared random effects can be interpreted as unmeasured factors that are related both to the longitudinal outcome and to mortality. Usually, an LME model is used for the longitudinal data and a proportional hazards model for the survival data, where the hazard for an event at time *t* is associated with the mean trajectory estimated via the LME model.

Whereas the GEE model estimates the average level of physical functioning among survivors, the joint model estimates the change in physical functioning. Joint models allow an investigator to assess, for example, how functioning would have developed in the original study sample if no one had died. Indirectly, these models can also evaluate developments among survivors only and answer questions about multiple outcomes, such as ‘what is the probability of being alive and having good functioning at 100 years of age?’. Especially in medical research, the joint modelling approach has gained great popularity since the introduction of Wu and Carroll’s (1988) model (Yu et al. 2017; Ahmadi-Abhari et al. 2017). With public releases of joint-model packages and commands for various software applications (e.g. JM, JMbayes and joineR in R; stjm in Stata; and JMfit in SAS), the availability of the approach in standard software has made it an even more attractive way of addressing dropout due to death.

## Method

### The Vitality 90+ Study

This study is based on the population-based Vitality 90+ Study, a multidisciplinary project with nonagenarians in Tampere, Finland (Jylhä et al. 2013). Vitality 90+ was undertaken to explore health, functioning, quality of life and their predictors among the oldest of the old (Sarkeala et al. 2011). The work reported here used Vitality 90+ data from five survey rounds in 2001, 2003, 2007, 2010 and 2014. Information on the target population (names, addresses, birth dates and places of residence) was obtained from the City of Tampere population register, which covers everyone whose official place of residence is in Tampere. All individuals in the area who were 90 or older in the study year were invited to participate (meaning that the youngest participants were 89), irrespective of health or place of residence. Response rates varied from 79 to 86% of those who were alive at the time of a given survey round, and the number of respondents increased from 892 in 2001 to 1637 in 2014, reflecting the rising number of the oldest old. According to Statistics, Finland (2018) people aged 90+ accounted for 0.44% of Tampere’s general population in 2001 and 0.85% in 2014. If the subject was unable to answer the questions, a family member or caregiver was asked to respond by proxy. The percentage of participants who took part by proxy varied from 15 to 23%.

### The study sample

**Fig. 1:**
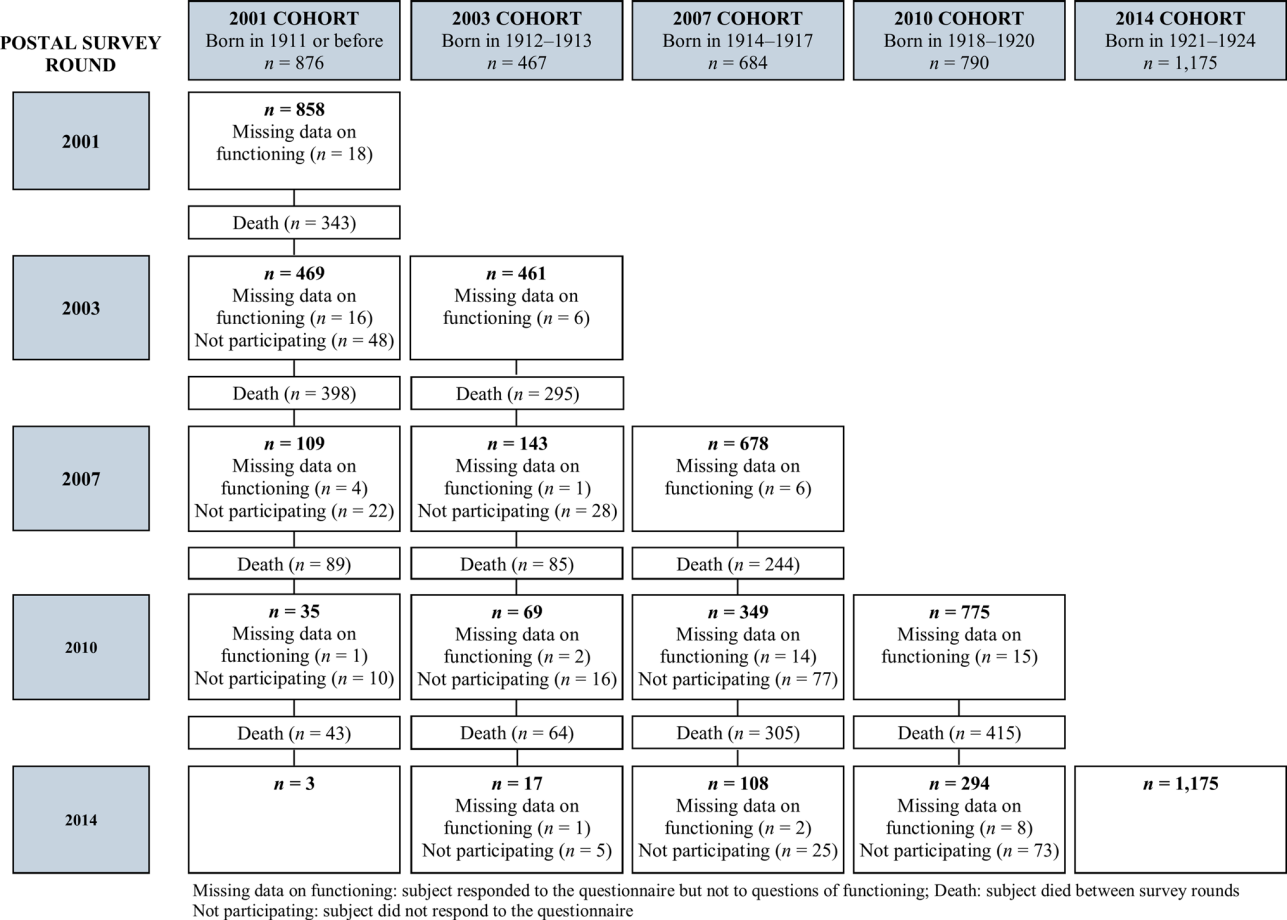
Flowchart for the study sample

To be included in our study, the participant had to have answered the questions on physical functioning at least once. This gave us a final sample of 3992 participants (3125 women and 867 men). The sampling process is presented in Fig. 1.

The cohort is defined by the year in which the subject completed the questionnaire for the first time. The 2001 cohort consists of all subjects born in 1911 or earlier (*n* = 876, age range 89–106 at the time of the questionnaire), with the subsequent cohorts bringing in only new individuals. Most participants responded only once (67.7%; of them 43.3% belonged to the 2014 cohort) or twice (25.5%); three people responded on all five survey rounds.

Death dates were obtained from Statistics Finland. Follow-up time was calculated from the date of the first questionnaire for each cohort to the common closing date, 31 May 2014, for survivors. The average follow-up time was 2.5 years (range 0.02–13 years). The study was approved by the Ethics Committee for Tampere Health Center or of Pirkanmaa Hospital District, depending on the year of data collection. All participants or a legal representative gave written informed consent.

### Measurement of the outcome, physical functioning

All rounds of the postal survey addressed physical functioning by asking participants whether they were able to perform five specific mobility and daily living activities: walk 400 metres, use stairs, move about indoors, dress and undress and get into and out of bed. The response options for each item were (1) ‘yes, without difficulty’; (2) ‘yes, with difficulty’; (3) ‘only with help’; and (4) ‘not at all’. For the calculation of a physical functioning score, answers were recoded as follows: 3 = ‘yes, without difficulty’; 2 = ‘yes, with difficulty’; ‘1 = only with help’; ‘0 = not at all’. The sum score ranged from 0 for those unable to perform any of the tasks, to 15 for those who indicated that they could manage all five without difficulty. The percentage of missing data on physical functioning caused by non-response varied from 0 to 4.3% between cohorts and survey rounds. Mortality (57%) remained by far the main reason for missing data points.

### Statistical analysis

In the case of GEE, we fitted the model

$$\begin{aligned} E(Y_{ij} ) & = \beta_{0} + \beta_{1} \times {\text{age}}_{ij} + \beta_{2} \times {\text{gender}}_{i} + \beta_{3} \times {\text{cohort}}2003_{i} \\ & \quad + \beta_{4} \times {\text{cohort}}2007_{i} + \beta_{5} \times {\text{cohort}}2010_{i} + \beta_{6} \times {\text{cohort}}2014_{i} \\ & \quad + \beta_{7} \times {\text{gender}}_{i} \times {\text{age}}_{ij} + \beta_{8} \times {\text{cohort}}2003_{i} \times {\text{age}}_{ij} \\ & \quad + \beta_{9} \times {\text{cohort}}2007_{i} \times {\text{age}}_{ij} + \beta_{10} \times {\text{cohort}}2010_{i} \\ & \quad \times {\text{age}}_{ij} + \beta_{11} \times {\text{cohort}}2014_{i} \times {\text{age}}_{ij} + \beta_{12} \times {\text{age}}_{ij}^{2} \\ \end{aligned}$$

where *E*(*Y*_*ij*_) represents the expected physical functioning score for subject *i* at the *j*th observation and where the *β*’s are the coefficients for intercept, age (continuous), gender (women as the reference group) and cohort (indicator variables for years 2003, 2007, 2010 and 2014, with 2001 as the reference). In all analyses, we used exact decimal age (i.e. the difference between the questionnaire completion date and date of birth), as *age*-*89*, to obtain a meaningful interpretation for the intercept term. To examine differences between men and women and between cohorts in changes over time, we also included interaction terms (gender × age and cohort × age) in the models. To allow for a nonlinear individual-level trajectory across time, an additional quadratic term for age was included. As Kurland et al. (2009) have recommended, we used a GEE model with an independent ‘working’ correlation structure for the modelling of physical functioning. With this correlation structure, the GEE model yields the same population-averaged estimates that the standard linear regression model does. Unlike the estimates of standard error from the standard linear regression which are biased due to correlation between subsequent measurements, the robust standard error estimator used with the GEE model yields standard errors, which are correct also for correlated longitudinal data. An IPW-GEE model was also fitted, but the difference between the estimates it produced and the GEE model’s output was negligible (results not shown).

In the case of LME, we fitted the model

$$\begin{aligned} Y_{ij} & = \beta_{0} + \beta_{1} \times {\text{age}}_{ij} + \beta_{2} \times {\text{gender}}_{i} + \beta_{3} \times {\text{cohort}}2003_{i} \\ & \quad + \beta_{4} \times {\text{cohort}}2007_{i} + \beta_{5} \times {\text{cohort}}2010_{i} + \beta_{6} \times {\text{cohort}}2014_{i} \\ & \quad + \beta_{7} \times {\text{gender}}_{i} \times {\text{age}}_{ij} + \beta_{8} \times {\text{cohort}}2003_{i} \times {\text{age}}_{ij} \\ & \quad + \beta_{9} \times {\text{cohort}}2007_{i} \times {\text{age}}_{ij} + \beta_{10} \times {\text{cohort}}2010_{i} \times {\text{age}}_{ij} \\ & \quad + \beta_{11} \times {\text{cohort}}2014_{i} \times {\text{age}}_{ij} + \beta_{12} \times {\text{age}}_{ij}^{2} + b_{i0} + b_{i1} \times {\text{age}}_{ij} + \varepsilon_{ij} \\ \end{aligned}$$

where *Y*_*ij*_ is the physical functioning score for subject *i* in the *j*th survey round, the *β*’s are the fixed-effect coefficients, and the *b* terms are the individual random-effect coefficients assumed to be distributed as (*b*_*i*0_, *b*_*i*1_)’ $\mathop \sim\limits^{i.i.d.} N\left( {0, \mathop \sum \nolimits_{b} } \right)$ and *ε*_*ij*_$\sim N\left( {0, \sigma_{\varepsilon }^{2} } \right)$ is the error of the *j*th observation for subject *i*. Fixed-effect coefficients determine the mean physical functioning score, and the random effects describe inter-individual variation around the mean. Stata/SE 15.1 for Windows was used to fit the GEE and LME models (with the commands xtgee and mixed).

Finally, in the case of the joint model, we used the R package JM developed by Rizopoulos (2010) to estimate the joint parameters for the longitudinal process and survival process. The longitudinal model for the physical functioning score was specified with an approach identical to the LME model. The physical functioning score was used as the outcome in the longitudinal part of the joint model, and its modelled mean was utilised as a covariate in the survival model. The other factors considered were gender and cohort. For the survival part of the joint model, a relative risk (proportional hazards) model was used (Rizopoulos 2012):

$$\begin{aligned} h_{i} \left( {t|M_{i} (t),{\text{ gender}}_{i} ,{\text{ cohort}}_{i} } \right) = & h_{0} (t ) {\text{exp}}\left\{ {\gamma_{1} \times {\text{gender}}_{i} + \gamma_{2} \times {\text{cohort}}2003_{i} } \right. \\ & \quad + \gamma_{3} \times {\text{cohort}}2007_{i} + \gamma_{4} \times {\text{cohort}}2010_{i} \\ & \quad \left. { + \gamma_{5} \times {\text{cohort}}2014_{i} - \alpha m_{i} (t)} \right\} \\ \end{aligned}$$

where *M*_*i*_(*t*) = {*m*_*i*_(*s*), 0 ≤ *s* < *t*} denotes the history of the individual-level physical functioning up to time point *t*, while the *γ* terms are the regression coefficients for gender and cohort. The baseline risk function, *h*_0_(*t*), is assumed to be piecewise constant with six knots, or dividing points, which are placed such that the number of observed events in the quantiles are nearly equal. The physical functioning and survival submodels are joined by the term *m*_*i*_(*t*), which is the individual-level-estimated physical functioning score at time *t*, and *α* represents its log hazard ratio. The above-mentioned joint model was fitted by using the R code supplied by Rizopoulos (2012), available also via http://jmr.r-forge.r-project.org/.

## Results

Summary baseline statistics for our study sample are shown in Table 1. Men accounted for about 20% of the first four cohorts and for 25% for the 2014 cohort. On average, participants in the first cohort (from 2001) were older because of how the study sample was defined: the first cohort in the study consisted of all individuals of aged 89 or over at the time of the questionnaire, whereas most respondents aged 92+ in 2003, for example, had already responded in 2001. Consequently, the 2001 cohort showed the lowest mean physical functioning figure at baseline. Nonetheless, 46% of the participants in all five cohorts appeared to be able to perform all five mobility and daily living activities, either without or with difficulty but still without help.

**Table 1:** Summary statistics for participants at the time of the first questionnaire: number (*n*) and proportion (%) of women and men and mean and standard deviation (SD) for age and physical functioning

Cohort	N	Female *n* (%)	Male *n* (%)	Age Mean (SD)	Physical functioning Mean (SD)
2001	876	707 (80.7)	169 (19.3)	92.2 (2.57)	9.4 (4.99)
2003	467	364 (77.9)	103 (22.1)	90.7 (1.36)	10.5 (4.65)
2007	684	541 (79.1)	143 (20.9)	91.8 (1.27)	10.1 (4.55)
2010	790	632 (80.0)	158 (20.0)	90.7 (1.46)	10.2 (4.53)
2014	1175	881 (75.0)	294 (25.0)	90.9 (1.34)	10.2 (4.58)

The parameter estimates for each model are presented in Table 2. Irrespective of the model used, men’s mean physical functioning score was significantly higher than women’s (by about 1.3 units) at 89 years of age.

**Table 2:** Results for physical functioning associated with gender, cohort and age from the generalized estimating equation (GEE), linear mixed-effects (LME) and joint models (for all models, the number of individuals (observations) is 3992 (5541))

	GEE	LME model	Joint model
Mean physical functioning for 89-year-olds in the 2001 cohort			
Women	10.0 (9.4, 10.6)	10.5 (10.1, 11.0)	11.4 (11.0, 11.9)
Men	11.3 (10.6, 12.0)	11.8 (11.3, 12.4)	12.7 (12.2, 13.3)
*P* value for difference between women and men	< 0.001	< 0.001	< 0.001
Mean physical functioning among 89-year-old women			
2001 cohort	10.0 (9.4, 10.6)	10.5 (10.1, 11.0)	11.4 (11.0, 11.9)
2003 cohort	11.0 (10.4, 11.6)	11.3 (10.8, 11.8)	11.6 (11.1, 12.1)
2007 cohort	10.8 (10.1, 11.4)	11.5 (10.9, 12.0)	12.3 (11.8, 12.8)
2010 cohort	10.5 (10.1, 10.9)	10.7 (10.3, 11.1)	11.0 (10.6, 11.4)
2014 cohort	10.7 (10.3, 11.2)	10.7 (10.2, 11.2)	10.7 (10.2, 11.2)
*P* value for difference between cohorts	0.055	0.012	< 0.001
Difference in slope for other cohorts compared to the 2001 cohort			
2003 cohort	− 0.17 (− 0.32, − 0.02)	− 0.18 (− 0.31, − 0.06)	− 0.16 (− 0.29, − 0.03)
2007 cohort	− 0.12 (− 0.29, 0.04)	− 0.18 (− 0.31, − 0.06)	− 0.20 (− 0.33, − 0.08)
2010 cohort	− 0.11 (− 0.29, 0.07)	− 0.06 (− 0.19, 0.08)	0.01 (− 0.12, 0.14)
2014 cohort	− 0.25 (− 0.48, − 0.01)	0.01 (− 0.22, 0.23)	0.21 (− 0.02, 0.44)
*P* value for difference in slope between cohorts	0.13	0.010	< 0.001
Average decline in physical functioning for each year of age for women	− 0.21 (− 0.41, − 0.004)	− 0.39 (− 0.54, − 0.24)	− 0.59 (− 0.73, − 0.44)
*P* value for age	0.046	< 0.001	< 0.001
Difference in slope for men compared to women	0.10 (− 0.03, 0.23)	0.08 (− 0.04, 0.19)	0.05 (− 0.06, 0.17)
*P* value for difference in slope between women and men	0.15	0.19	0.37
Quadratic term for age: (age-89)^2^	− 0.01 (− 0.02, 0.01)	− 0.02 (− 0.03, − 0.01)	− 0.03 (− 0.04, − 0.02)
*P* value for quadratic term for age	0.20	< 0.001	< 0.001

Because the participants in the 2001 cohort were on average older than the other cohorts and exhibited lower average physical functioning scores, significant differences are visible between cohorts with the LME model (*P *= 0.012) and the joint model (*P* < 0.001). The change in physical functioning score (that is, the difference in slope for other cohorts compared to the 2001 cohort, Table 2) also differed between cohorts in two of the models (LME model: *P* = 0.010; joint model: *P* < 0.001). Greater declines compared to the 2001 cohort were observed in the 2003 and 2007 cohorts, which also had higher baseline physical functioning. A similar pattern was suggested by the GEE, but these differences were not overall statistically significant (*P* = 0.13).

As expected, we found significant age effects with all of the models: physical functioning declines as a function of age (see Table 2). However, the differences between the models were clear: they yielded widely differing estimates of the rate of decline in physical functioning. The linear estimate for decline was borderline significant in the GEE case, one-third of that shown by the joint model, which represented the largest decline found. The LME estimate fell in between the two.

**Fig. 2:**
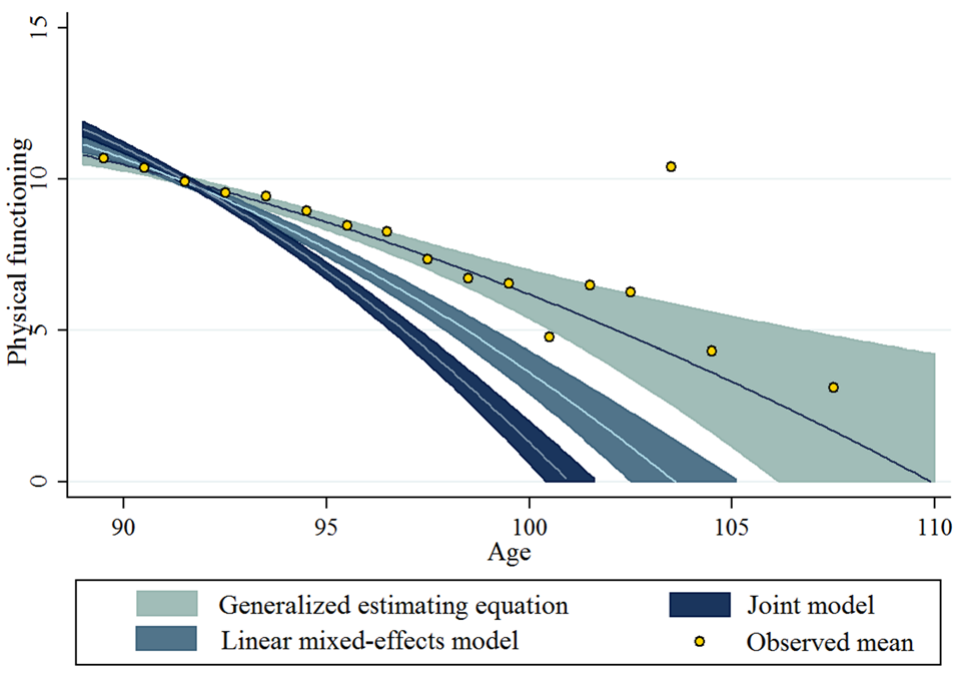
Average physical functioning score trajectories and their 95% confidence bounds under the generalized estimating equation model, the linear mixed-effects model and the joint model

The results indicate, then, that the average individual decline was remarkably faster than the decline among the survivors. This was particularly so for men: according to the joint model estimate the individual linear part of the decline was approximately five-fold (− 0.59 + 0.05 = − 0.54, Table 2) compared to that of the decline among survivors (− 0.11). The quadratic term for age shows that the rate of change in physical functioning was not constant. For example, the average decline rates (gradients) for an 89-year-old woman in the 2001 cohort were 0.21 with the GEE model and 0.59 with joint modelling. The corresponding rates for a 95-year-old woman were 0.33 and 0.95 units. Change in physical functioning was thus not linear, but it showed acceleration with each additional year of age. The magnitude of the estimated acceleration in decline also appeared to differ between models, achieving significance with the LME and joint model but not with the GEE model. Fitted average physical functioning score trajectories in Fig. 2 provide graphic representations of the results from the three models.

We observed large variation at the individual level as assessed by the random intercept term of the LME and the joint model. The estimates of variability were similar: standard deviation in the LME model was 2.43 and in the joint model 2.28. However, the standard deviation for the regression coefficients on age was 0.34 in the LME and 0.47 in the joint model, suggesting that inter-individual variation in the decline rate was estimated to be almost 40% higher in the joint model than in the LME.

In the joint model for the survival process (see Table 3), the parameter that measures the association between physical functioning score and risk of death (*α*) can be expressed as the hazard ratio for physical functioning. A strong association emerged, with every one-unit decrease in the physical functioning score corresponding to a 1.17-fold increase in the risk of death (95% confidence interval 1.16–1.19).

**Table 3:** Hazard ratios (HRs) with 95% confidence intervals (CIs) for the survival process in the joint model

	HR (95% CI)
Gender (ref. = women)	1.72 (1.52, 1.92)
Cohort (ref. = 2001 cohort)	
2003	1.26 (1.11, 1.43)
2007	0.99 (0.89, 1.12)
2010	1.19 (1.04, 1.34)
2014	0.24 (0.18, 0.34)
One-unit decrease in physical functioning score	1.17 (1.16, 1.19)

## Discussion

The current study compared the findings and characteristics of three distinct methods in analysing change in physical functioning among the oldest old over a follow-up period of a maximum of 13 years. In the population-based sample of people aged 90 and over from the Vitality 90+ Study, we found that physical functioning clearly declined with age, and that the decline in physical functioning accelerated with advancing age. For both the rate of decline in physical functioning and its acceleration, we found that the results differed markedly between the three distinct models—GEE, LME, and joint modelling—both numerically and in conceptual terms. The average individual decline in physical functioning derived from individual trajectories via the LME and the joint model was clearly sharper than the average decline among survivors estimated with GEE modelling. This result is consistent with earlier findings based on a younger population sample (Jones et al. 2015) and with a binary outcome (Chang et al. 2009).

We found that the results differed substantially between the three models, most clearly so between the GEE one and the joint model. The most important underlying factor was dataset incompleteness caused by high mortality, which meant that a large proportion of participants died during each two-to-four-year interval between survey rounds. Nearly 70% of the participants responded only once because they either died before the next survey or, in the case of those enrolling in 2014, had only one chance to participate. As a consequence of high missingness through death, the estimate of average individual-level decline differed markedly from the estimate of decline among those who remained alive.

GEE modelling, accounting for the varying number of subjects who were alive in each survey round and producing an estimate of the population mean among them, indicated clearly less decline in physical functioning with age than the other two models. This is understandable in that the individuals who participated were likely to show better physical functioning than those who died between surveys or who were so close to death that they no longer took part. In our LME model, the longitudinal trajectory, modelled as a curve for the mean and for each subject, compensates only partially for the missing data, i.e. it offsets the absence of data only to the extent that it can be attributed to and explained by the observed values and the components incorporated into the model (those components being the actual observations for physical functioning, cohort and gender). Accordingly, LME modelling is valid for individual and population trajectories only if one can assume that the dropout is of the ‘missing at random’ type (Sterne et al. 2009). The joint model, on the other hand, fully corrects for death while also performing the corrections made with the LME model. Therefore, in our data joint modelling showed a remarkably steep decline in physical functioning with age compared to that found by the LME model alone, and both were steeper than the result with the GEE model. This implies that an average individual suffers from a rapid and accelerating decline in physical functioning after age 90, and this decline is considerably steeper among those who are close to death than those who still have more years to live. This is consistent with earlier findings which showed a decrease in physical functioning with increasing proximity to death, but also decreasing physical functioning with increasing age (Guralnik et al. 1991). Yet there are practically no earlier findings on age-related changes in functioning specifically among the oldest old.

Given the continuing increase in life expectancy—in Finland, for example, life expectancy at 90 grew from 3.4 to 3.8 years for men and from 3.9 to 4.4 years for women between 2001 and 2014 (Statistics Finland 2018)—it is of paramount importance to understand how functioning develops in the oldest segment of the population. For those interested in the impact of changes in the age structure on functioning and the situation within the older portion of the relevant population, GEE models may be useful. But if one is interested in the ageing process itself and in the consequences of increasing age for physical functioning, then joint models are likely to yield the most appropriate information. In our study, we found that regardless of the model used, each additional year beyond the age of 90 brought increasing problems with physical functioning. Because of their ability to account for longitudinal trajectories and death, joint models reveal a steeper and accelerating trend of functional decline with age than other models do. Estimated slopes for dying participants were steeper in the joint model compared to LME and, hence, the variability of the random regression coefficients on age was greater in the joint model than in LME. We used the joint model primarily to adjust longitudinal measurements with dropout due to death, but the joint model can also be used to study the distribution of time to death conditional on intermediate longitudinal measurements, or the joint development of the measurement and survival processes (Henderson et al. 2000).

The strengths of the study described here include the exceptionally large longitudinal dataset for very old individuals, comprising nearly 4000 participants, a high response rate and identical study design and survey questions in each survey round. Because as many as 57% of the participants died during the follow-up, this constitutes an ideal body of data for purposes of comparing longitudinal models that address dropout due to death. The greatest limitation of this study is that on average, the questionnaire was administered only once every 3.25 years: the high death rate meant that most participants were able to answer only once. Of them, 43.3% belonged to the 2014 cohort. This cohort differs markedly from the other cohorts also in that the follow-up time was only four months compared to a 4–13 year in four other cohorts. It is likely that those people close to death never became participants of the 2014 cohort, which could cause underestimation of its hazard ratio. Accordingly, these subjects, while contributing cross-sectional data and influencing estimates of the average level of physical functioning, had much less weight in the results for changes in physical functioning over time.

In conclusion, the estimates for change in physical functioning over 13-year follow-up differed considerably between the models and depended greatly on whether or not the modelling accounted for dropout due to death. It is therefore crucial to have a thorough knowledge of the various longitudinal methods of analysis so that one can choose the model that is best suited to the research question. Among the methods available, LME displays the least appropriate handling of dropout due to death. Ignoring this dropout at the modelling stage may lead to biased estimates and therefore to inappropriate conclusions. The GEE and joint models, on the other hand, are both valid. They may, however, yield substantially different results because they are directed at different population elements and thus answer different research questions. A GEE model may be more useful for considering the effects of intervention measures on the outcome among the living. Joint models, for their part, may be valuable for predicting the needs of long-living individuals whose functional status can rapidly change, and for planning elderly care for populations whose death is rapidly shifting to older ages.
